# The lncRNAs/miR-30e/CHI3L1 Axis Is Dysregulated in Systemic Sclerosis

**DOI:** 10.3390/biomedicines10020496

**Published:** 2022-02-19

**Authors:** Valentin Dichev, Nikolay Mehterov, Maria Kazakova, Rositsa Karalilova, Anastas Batalov, Victoria Sarafian

**Affiliations:** 1 Department of Medical Biology, Medical University-Plovdiv, Blvd. 15A Vasil Aprilov, 4002 Plovdiv, Bulgaria; valentin.dichev@mu-plovdiv.bg (V.D.); mariya.kazakova@mu-plovdiv.bg (M.K.); victoria.sarafian@mu-plovdiv.bg (V.S.); 2 Research Institute, Medical University-Plovdiv, Blvd. 15A Vasil Aprilov, 4002 Plovdiv, Bulgaria; 3 Department of Propedeutics of Internal Diseases, Medical University-Plovdiv, Vasil Aprilov Blvd. 15A, 4001 Plovdiv, Bulgaria; rositsa.karalilova@mu-plovdiv.bg (R.K.); anastas.batalov@mu-plovdiv.bg (A.B.); 4 Clinic of Rheumatology, University Hospital “Kaspela”, 64 Sofia Str., 4001 Plovdiv, Bulgaria

**Keywords:** MALAT1, NEAT1, miR-30e, systemic sclerosis, CHI3L1

## Abstract

Systemic sclerosis (SSc) is an autoimmune disease with completely undefined etiology and treatment difficulties. The expression of both protein coding and non-coding RNAs is dysregulated during disease development. We aimed to examine a possible regulatory axis implemented in the control of chitinase-3 like protein 1 (CHI3L1) or YKL-40, an inflammation-associated glycoprotein, shown to be elevated in SSc. A panel of seven miRNAs and three lncRNAs potentially involved in the control of CHI3L1 were selected on the basis of in silico analysis. TagMan assay was used to evaluate the expression levels of miRNAs and RT-qPCR for lncRNAs in white blood cells (WBCs) and plasma from SSc patients and healthy controls. Among the eight screened miRNAs, miR-30e-5p (*p* = 0.04) and miR-30a-5p (*p* = 0.01) were significantly downregulated in WBCs and plasma of SSc patients, respectively. On the contrary, the expression of the metastasis associated lung adenocarcinoma transcript 1 (MALAT1) (*p* = 0.044) and the Nuclear enriched abundant transcript 1 (NEAT1) (*p* = 0.008) in WBCs was upregulated compared to the controls. Increased levels of MALAT1 and NEAT1 could be associated with the downregulation of miR-30e-5p and miR-30a-5p expression in WBCs and plasma. We present novel data on the involvement of a possible regulatory axis lncRNAs/miR-30e/CHI3L1 in SSc and hypothesize that MALAT1 and NEAT1 could act as miR-30e-5p and miR-30a-5p decoys. This may be a reason for the increased serum levels of CHI3L1 in SSc patients.

## 1. Introduction

Systemic sclerosis (SSc) is an autoimmune disease with unclear etiology, which poses treatment difficulties. The pathological findings include skin and internal organ fibrosis, vascular injury and capillary thrombosis, and a chronic autoimmune inflammatory response [1]. The links between these processes are numerous signaling molecules with pro- and anti-inflammatory and pro- and anti-fibrotic effects. Their levels are usually imbalanced, creating a self-sustaining cycle of damaging processes. They result in excessive fibroblast activation and deposition of components of the extracellular matrix, which lead to impaired quality of life and shorter life expectancy [2]. Usually, the treatment is not efficient enough, and the lack of reliable markers for its development and prognosis, makes SSc management challenging. Along with the expression of cytokines and other proteins, non-coding RNAs which play role in epigenetic regulation, are also dysregulated during disease development, thus affecting its pathophysiological mechanisms [3]. These molecular imbalances suggest that the levels of both proteins and RNA molecules could serve as biomarkers during the disease course.

Epigenetic regulation plays an important role in the normal, as well as in the pathological state of cells and of the organism as a whole. It is proposed to drive or at least to accompany some autoimmune diseases [4]. Among the most common epigenetic regulators, the action of non-protein coding RNAs has attracted serious attention in the past few years. Some of the best characterized non-coding RNA molecules are the long non-coding RNAs (lncRNA), which are more than 200 nucleotides in length and usually possess a hair-loop structure, and microRNAs (miRNA), that are typically 22–25 nucleotides long [5]. These RNA molecules affect gene expression in a different mode.

LncRNAs function on several levels through various mechanisms, such as interaction with chromatin and DNA, leading to either gene silencing or activation. Moreover, they participate in chromosome scaffolding and condensation, post-transcriptional regulation through interaction with different proteins and miRNA sponging [6]. The mode of action of lncRNAs suggests that they are either positive or negative gene expression regulators. MiRNAs, on the other hand, have a negative effect on the realization of genetic information, as their role in epigenetic regulation is to decrease protein synthesis by blocking translation or by directly targeting mRNA for degradation [7]. In addition, miRNAs could be detected in the circulation as single molecules, in complexes with proteins, or in exosomes. In this way, they behave as endocrine factors, which affect gene expression in distant cells and tissues [8].

CHI3L1 or YKL-40 is a secreted glycoprotein with a pleiotropic function. It is synthesized by different cell types like activated macrophages, neutrophils, fibroblasts, chondrocytes, endothelial, synovial and various tumor cells [9]. The exact function of CHI3L1 is not determined yet but it is known to possess pro-fibrotic, pro-angiogenic and pro-metastatic effects. The protein is involved in both acute and chronic inflammation, through binding to several receptor molecules [10]. The CHI3L1 protein is released in all body fluids and its levels are increased in serum of SSc patients and in other autoimmune rheumatic diseases [11]. Our previous studies showed increased concentrations of CHI3L1 in sera of SSc patients with no difference in CHI3L1 mRNA expression in white blood cells (WBCs) of patients and healthy controls. Additionally, we found that miR-214, which retains a binding site for CHI3L1 mRNA 3’UTR, is decreased in patients’ plasma. These results underpin the speculation for the existence of a regulatory mechanism for CHI3L1 synthesis, based on interactions of lncRNAs and miRNAs [12].

Therefore, the aim of the present study was to examine a possible regulatory axis through lncRNAs and miRNAs, implemented in the control of CHI3L1 expression in SSc. First, a panel of seven miRNAs and three lncRNAs feasibly involved in the control of CHI3L1 were selected on the basis of in silico analysis. Among the considered miRNAs that might target the CHI3L1 mRNA, miR-30e-5p was downregulated in WBCs and miR-30a-5p was decreased in plasma of SSc patients, which is in accordance with increased glycoprotein levels. The bioinformatical analysis showed that MALAT1 and NEAT1 have binding sites for both miRNAs, suggesting that they can act as miRNA decoys. This was further supported by the increased levels of MALAT1 and NEAT1 in WBCs. Additionally, miR-30a-5p plasma levels could be used as a biomarker to distinguish SSc patients from healthy individuals. The current study revealed the possible existence of a lncRNAs/miR-30e/CHI3L1 regulatory axis in SSc.

## 2. Materials and Methods

### 2.1. Participants

The clinical characteristics of the SSc patient cohort and healthy volunteers were published previously [12]. Briefly, 40 female patients and 14 healthy age-matched female subjects were enrolled in the present study. Patients were diagnosed with SSc in accordance to ACR/EULAR 2013 criteria [13] and subtyped as diffuse cutaneous SSc (dcSSc) and limited cutaneous SSc (lcSSc) according to the classification criteria of LeeRoy and Medsger, based on the extension of skin fibrosis. [14]. Healthy participants were recruited based on exclusion criteria: age below 18 years; presence of malignancies, inflammatory joint and other autoimmune diseases. The study was approved by the Ethics Committee of Medical University-Plovdiv (protocol No. 3/31.05.2018). No difference in age distribution was observed between controls and patients, as well as between the disease subgroups (dcSSc, lcSSc). Comparison between different hematological, serological and clinical parameters like ESR, CRP, ANA positivity, disease duration, capillaroscopy, skin thickening was assessed by the modified Rodnan skin score (mRSS). Internal organ involvement was evaluated and corresponding treatment programs were performed. The clinical data and demographic characteristics of the patients are available in Supplementary Table S1.

### 2.2. Plasma, Serum and White Blood Cells (WBCs) Isolation

Blood samples were taken between 8:00 and 9:00 a.m. on the day of admission of each patient. The sampling of healthy volunteers was tailored to the protocol applied for patients. Venous blood samples were obtained through venipuncture in vacuum containers (BD Vacutainer^®^) from each individual. After centrifugation at 3000 rpm for 10 min, plasma and blood cells were separated. Plasma was aliquoted and stored at −80 °C, for further RNA isolation. Erythrocytes were lysed with an ammonium chloride based lysis buffer (NHCO3 3.4 mM, NH4Cl 155 mM, EDTA 96.7 µM). The pellet of leukocytes in which neutrophils, monocytes, lymphocytes and eosinophils were present, was used for total RNA extraction. Serum was obtained from vacuum tubes with clot activator following centrifugation at 3000 rpm for 10 min and stored at −20 °C, for further analysis.

### 2.3. RNA Isolation from Plasma/WBC and DNase Treatment

Plasma samples from each individual were used for isolation of circulating extracellular RNA, by the NORGEN Plasma/Serum RNA Purification midi kit (Biotek Corp, Thorold, ON, Canada, Lot. No. 56100), following the manufacturer’s instructions. After thawing, plasma samples were centrifuged for removing debris. Lysis buffer A and isopropanol were then added and the plasma homogenate was passed by centrifugation through the Midi Spin column. Three washing steps with wash solution A were carried out and elution buffer F was applied to the column to extract RNA. Lysis buffer A and 100% ethanol were added to the eluted RNA. Then, the mixture was passed through the Mini Spin column by centrifugation. The following washing and elution with Wash solution A and Elution solution A were subsequently performed. Eluted RNA was stored at −80 °C, for further analysis.

TRIzol Reagent (Thermo Fisher Scientific, Waltham, MA, USA, Lot. No. 1559602,) was applied to extract total RNA from WBC. In brief, chloroform was added to TRIzol homogenized cells. After centrifugation, the aqueous phase, containing total RNA was carefully transferred to a new tube, precipitated with isopropanol and then washed with 75% ethanol. Finally, RNA was solubilized in DNAase/RNAase free water. Following RNA extraction, samples were treated with a TURBO DNA-free kit (Thermo Fisher Scientific, Waltham, MA, USA, Lot. No. AM1907,) to remove residual DNA. Shortly after, RNA was mixed with 3 μL TURBO DNase™ Buffer and 1.5 μL TURBO DNase™ Enzyme. After 50 min. incubation at 37 °C, DNase Inactivation Reagent was added. Subsequently, samples were centrifuged and the supernatant containing the RNA was obtained. The exact RNA concentrations were determined spectrophotometrically by NanoDrop 2000 (Thermo Fisher Scientific, USA).

### 2.4. ELISA

Evaluation of serum YKL-40 protein levels in all SSc patients and controls was performed by commercial ELISA kit, specific for the protein encoded by the longer isoform (383 amino-acids) (MicroVue YKL-40, Quidel, 9975 Summers Ridge Road, San Diego, CA 92121, USA, Lot. No. 088337), following the manufacturer’s instructions. The assay employs the sandwich based ELISA method with optical density measured at 405 nm. Based on OD results, concentrations of standards, controls and samples were determined. The detection rate of the kit is 5.4 ng/mL and the within- and between-run CV are 5.8 and 6.0%, respectively. All samples were examined in duplicate.

### 2.5. miRNA:mRNA and lncRNA:miRNA Target Prediction

Identification of potential miRNAs targeting the 3’-untranslated region (UTR) of CHI3L1 was performed by three prediction tools: microRNA.org [15], TargetScan (http://www.targetscan.org/vert_80/ (17 January 2021) [16] and miRmap (https://mirmap.ezlab.org/ (17 January 2021) [17]. All predicted miRNAs were selected for transcription analysis. DIANA-LncBase v3 (https://diana.e-ce.uth.gr/lncbasev3/home (23 January 2021) [18,19] and Starbase (https://starbase.sysu.edu.cn/ (23 January 2021) [20] bioinformatic tools were used for lncRNA- miR-30c/30e target prediction. These tools are designed for the identification of putative miRNA:mRNA or lncRNA:miRNA interactions, depending on base-pair complementarity and target prediction score.

### 2.6. Reverse Transcription and qPCR

Two μg total RNA were reverse transcribed by the RevertAid H Minus First Strand cDNA Synthesis Kit (Thermo Fisher Scientific, Waltham, MA, USA, Lot. No. 00648151) and the obtained cDNA was used for the quantitative measurement of CHI3L1, MALAT1, NEAT1 and UCA-1 expression. Following that, the cDNA was used as a template for amplification in a quantitative PCR reaction by Genaxon GreenMasterMix (2x) (Genaxxon bioscience GmbH, Ulm, Germany, Lot. No. M3023.0500) following the manufacturer’s recommendations. Specific primers for the longest RNA transcripts of CHI3L1 (ENST00000255409.8, the sequence coding the functional 383 amino-acid long CHI3L1 protein) (Fw 5’-CTGCTCCAGTGCTGCTCT-3’, Rev 5’-TACAGAGGAAGCGGTCAAGG-3’), MALAT1 (ENST00000619449.2, Fw 5’-GCCTCAGCTCGCCTGAAGGTG-3’ Rev 5’-TCAATGCCTACCGCACAGCTCGG-3’), NEAT1 (ENST00000501122.2, Fw 5’-ATCCAGGGTCCTCTTTCTGC-3’ Rev 5’-AGGCGGTTATAGAGGTGCTG-3’) and UCA1(ENST00000645805.1, Fw 5’-TTTGCGTCACCTCAGTGAAGGTGG-3’ Rev 5’-TCCTGTAGGCCACCTCTCTGGTC-3’). The obtained expression values were normalized to the levels of GAPDH (Fw 5’-AGG TCCACCACTGACACGTTG-3’, Rw 5’-AGCTGAACGGGATGCTCACT-3’), ACTINβ (Fw 5’-AGTGTGACG TGGACATCCGGA-3’, Rev 5’-GCC AGGGCAGTGATCTCCTCCT-3’) and hUBC (Fw 5’TCCTCAGGCAGAGGTTGATCTT-3’, Rev 5’-GGACCAAGTGCAGAGTGGACTCTT-3’) (Integrated DNA Technologies, Leuven, Belgium). The qPCR reactions were run on Rotor-Gene Q 600 (Qiagen, Germany) and the relative expression of CHI3L1, MALAT1, NEAT1 and UCA-1 was normalized to the geomean of the three housekeeping genes using the comparative 2^−ΔΔCt^ method. All samples were analyzed in duplicates.

### 2.7. TagMan Analysis

TagMan MicroRNA Reverse Transcription Kit (Thermo Fisher Scientific, Waltham, MA, USA, Lot. No. 4366596) and TagMan MicroRNA Assays primers (Thermo Fisher Scientific, Waltham, MA, USA, Lot. No. 4427975) were used for the quantitative measurement of miR-24-3p, miR-30a-5p, miR-30b-5p, miR-30c-5p, miR-30e-5p, miR-30d-5p, miR-125a-3p expression in WBCs and plasma. snoRNA U48 and U6 were used as endogenous controls. cDNA specific for all predicted miRNAs, as well as for snoRNA U48 and U6 was reverse transcribed by specific stem-loop 5x primers. Next, 5 µL cDNA (1:7 diluted) was used as a template in TagMan PCR reaction for miRNA gene expression levels evaluation. In addition to cDNA, each reaction contained 5.03 µL TagMan Advanced master mix (Thermo Fisher Scientific, Waltham, MA, USA, Lot. No. 00692208) and 0.47 µL miRNA 20x primers in a total volume of 10.5 µL. All PCR reactions were run on Rotor-Gene Q 600 machine (Qiagen, Hilden, Germany). Ct values of each analyzed miRNA were normalized to snoRNA U48 in WBCs and to U6 in plasma, respectively. All reactions were performed in duplicates.

### 2.8. Statistical Analysis

Quantitative data were analyzed using the GraphPad Prism software (GraphPad Software 8.0.1 version, Inc., La Jolla, CA, USA). Distribution was assessed by DÁgostino & Pearson, Shapiro–Wilk and Kolmogorov–Smirnov test’s. To compare protein levels and FC values between the different groups, Student’s two-tailed t-tests or Mann–Whitney U tests were performed. Parametric and non-parametric data were presented as mean (SD) and median (minimum–maximum), respectively. The diagnostic potential of plasma miRNAs was accessed by receiver operating characteristic (ROC) curves and the area under the curves (AUCs) was calculated. The threshold for significance was set at 0.05 (*p* > 0.05, indicated with *), *p*-values less than 0.01 are marked with **.

## 3. Results

### 3.1. miR-30a-5p and miR-30e-5p Are Downregulated in SSc

**Figure 1:**
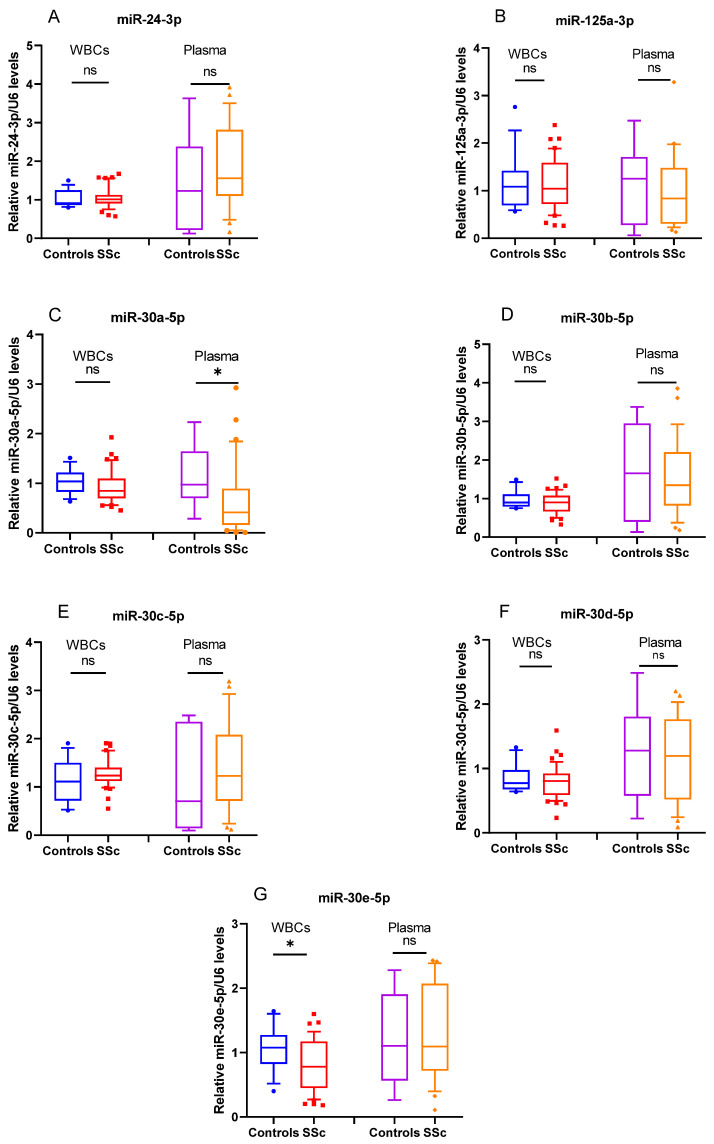
miR-30a-5p and miR-30e-5p are downregulated in SSc. Box plot of (**A**) miR-24-3p, (**C**) miR-30a-5p, (**D**) miR-30b-5p, (**E**) miR-30c-5p, (**G**) miR-30e-5p, (**F**) miR-30d-5p and (**B**) miR-125a-3p measured in both WBC and plasma of SSc (WBC, *n* = 31; plasma, *n* = 30) and healthy controls (WBC, *n* = 12; plasma, *n* = 9). Expression levels were determined by TagMan assay after total RNA extraction and presented as fold difference. snoRNA U48 and U6 were used as miRNA normalizers. The data are summarized from two technical replicates for each examined individual. *p* > 0.05 was marked with *.

Previously we have shown that serum protein levels of YKL-40 in SSc were significantly increased in comparison to healthy subjects. In addition, concentrations of the glycoprotein were higher in patients with dcSSc compared to those with the limited form of the disease. We suspect that the raised protein concentrations are due to a disbalance in the mechanisms regulating its expression. Moreover, elevated ESR and mRSS levels were observed in the dcSSc group. This result was related to the more severe inflammation and fibrosis detected in these patients [12]. In order to test whether CHI3L1 could be regulated by different epigenetic mechanisms, such as miRNAs, we employed targeting algorithms (TargetScan, miRmap and microRNA.org) to search for putative miRNAs that might bind to CHI3L1 mRNA. The in silico analysis of the 3’ UTR region of CHI3L1, revealed several potential miRNAs binding sites for seven miRNAs, including miR-24-3p, miR-30a-5p, miR-30b-5p, miR-30c-5p, miR-30e-5p, miR-30d-5p and miR-125a-3p [12]. Based on the results from the in silico analysis, a TagMan assay was applied to analyze each of the seven miRNAs in SSc patients and healthy subjects. Their expression pattern in healthy controls and SSc patients is presented in Figure 1. Among the analyzed miRNAs, the transcriptional levels of miR-30e-5p (*p* = 0.03) (G) and miR-30a-5p (*p* = 0.01) (C) were found to be significantly downregulated in SSc WBC and plasma, respectively.

**Figure 2:**
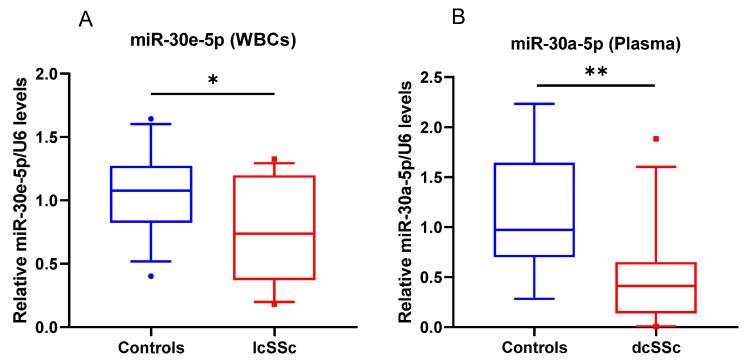
miR-30e-5p and miR-30a-5p are differentially expressed in SSc subgroups. Box plot of miR-30e-5p (**A**) in WBCs of lcSSc patients (*n* = 12) and miR-30a-5p (**B**) in plasma of dcSSc patients (*n* = 14), compared to healthy controls (WBC, *n* = 12; plasma, *n* = 9). Expression levels were determined by TagMan assay after total RNA extraction and presented as fold difference. snoRNA U48 and U6 were used as miRNA normalizers. The data are summarized from two technical replicates for each examined individual. *p* > 0.05 was marked with *; *p* > 0.01 was marked with **.

Additionally, miR-30e-5p was decreased in WBCs of patients with lcSSc (*p* = 0.04) and miR-30a-5p was considerably lower in plasma of dcSSc patients (*p* = 0.005) compared to controls (Figure 2). Following these results, we suggest that the downregulation of miR-30e-5p and miR-30-5p in SSc could be a reason for the high protein levels of CHI3L1 in serum.

### 3.2. MALAT1 and NEAT1 Are Upregulated in SSc WBCs

**Figure 3:**
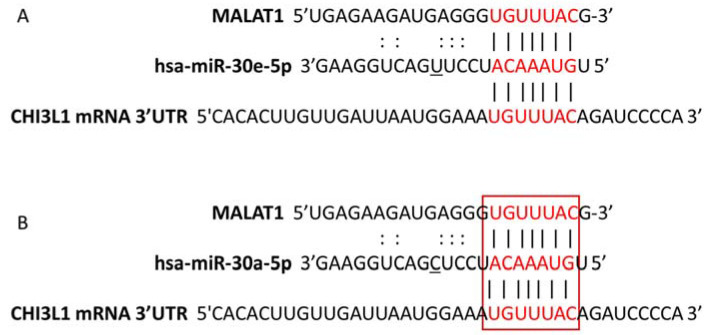
Binding sites of miR-30e-5p (**A**) and miR-30a-5p (**B**) in CHI3L1mRNA and MALAT1. DIANA-LncBase v3 and Starbas bioinformatical tools were applied to predict the binding sequence of the two down regulated miRNAs: miR-30e-5p and miR-30a-5p in the 3’UTR of CHI3L1mRNA and lncRNA MALAT1. The in silico analysis revealed that both have a conserved seed region by which they could bind to the complementary sites in CHI3L1mRNA and lncRNA MALAT1 molecules.

LncRNAs are proven to act as miRNA decoys, thus indirectly modulating the expression of many protein-coding genes. In our previous work, we have shown that there was not any difference in CHI3L1 mRNA expression in the examined groups. However, there was an obvious increase in serum CHI3L1 protein levels [12]. Additionally, in the present study, we found that miR-30a-5p and miR-30e-5p, two miRNAs with 3’UTR CHI3L1 binding sites, were downregulated in SSc. Following this, we hypothesized that certain lncRNAs may sponge miR-30e-5p and miR-30a-5p, leading to increased CHI3L1 production. To address this, a follow up in silico analysis was performed in order to predict possible interactions between miR-30a-5p/miR-30e-5p and lncRNAs. Results from bioinformatics screening by the use of DIANA-LncBase v3 and Starbas tools revealed three lncRNAs–MALAT1, NEAT1 and UCA1, having binding sites for both miRNAs. Predicted binding sites for miR-30e-5p (A) and miR-30a-5p (B) in MALAT1 and CHI3L1 mRNA are shown in Figure 3.

**Figure 4:**
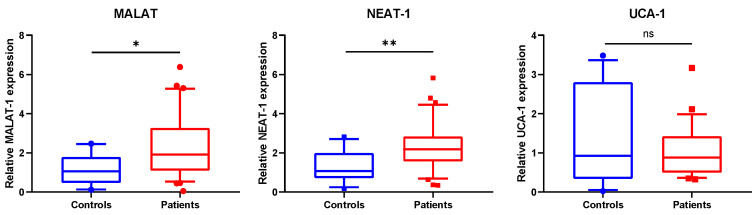
MALAT1 and NEAT1 are upregulated in SSc WBCs. MALAT1 and NEAT1 are induced in WBCs of SSc patients, compared with the control subjects. Expression levels of MALAT1, NEAT1 and UCA1 were evaluated in WBCs of patients (*n* = 31) and controls (*n* = 12) by RT-qPCR after total RNA extraction and presented as fold difference. GAPDH, ACTINβ and hUBC were used as RNA normalizers. The data are summarized from two technical replicates for each examined individual. *p* > 0.05 was marked with *; *p* > 0.01 was marked with **.

This finding together with the suggested involvement of MALAT1, NEAT1 and UCA1 in inflammation was the reason for their selection for further analysis [21,22,23]. Our results clearly indicated that among the three lncRNAs investigated, the expression levels of MALAT1 and NEAT1 were significantly changed. MALAT1 (*p* = 0.044) and NEAT1 (*p* = 0.008) were upregulated in SSc patients compared to controls (Figure 4).

### 3.3. miR-30a-5p Plasma Levels as a Discriminatory Marker in SSc

**Figure 5:**
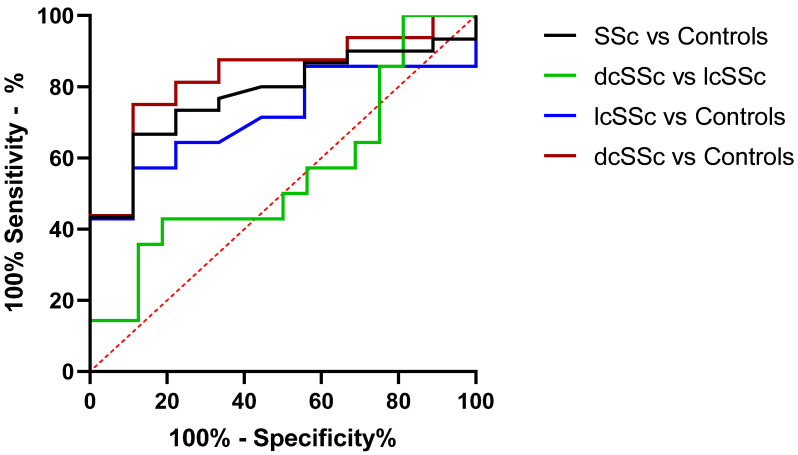
Discriminatory potential of plasma miR-30a-5p levels. ROC analysis was performed to access the discriminatory power of miR-30a-5p in plasma. The AUC values show that miR-30a-5p has the potential as a biomarker for distinguishing SSc from healthy subjects.

Secretion of miRNAs in the circulation justifies their usage as non-invasive biomarkers. To determine whether the downregulation of miR-30a-5p can distinguish patients with SSc from healthy controls, an ROC curve was constructed to evaluate the discrimination power of these miRNAs as potential biomarkers for SSc diagnosis. We found that miR-30a-5p expression in blood of SSc patients has the potential to discriminate dcSSc patients from healthy subjects (AUC = 0.83; *p* = 0.007), as well as the entire group of SSc patients from controls (AUC = 0.78; *p* = 0.01) (Figure 5). Our results show that miR-30a-5p plasma levels could be used as a biomarker in SSc.

## 4. Discussion

Autoimmune diseases, such as SSc present with complex pathological features and have an enigmatic etiology. Increased or decreased production of numerous molecules, implemented in diverse but related processes, like fibrosis, vasculopathy and inflammation are believed to be of great importance for disease development and progression. These changes could be a result of alterations in mechanisms, such as posttranslational regulation by lncRNAs and miRNAs [24]. Data concerning the involvement of miRNAs in autoimmunity are updated annually, while information about the role of lncRNAs is still elusive [25,26]. Here, we present novel information about the dysregulation of two lncRNAs MALAT1 and NEAT1, that may affect the expression of miR-30e-5p, which results in raised serum protein levels of CHI3L1 in SSc. The in silico analysis shows that the 3’UTR of CHI3L1, as well as part ofMALAT1 and NEAT1, share similar sequences allowing the binding of miR-30 family members. This suggests the possible existence of a regulatory mechanism between these RNA molecules. We have previously described that serum concentrations of the glycoprotein are higher in patients with SSc compared to healthy subjects. A significant difference is also observed in individuals with dcSSc when matched with those with lcSSc [12,27].

LncRNA MALAT1 is shown to play role in oncogenesis and cancer development. Information about its involvement in the pathophysiology of autoimmune diseases, such as rheumatoid arthritis (RA) and Systemic lupus erythematosus (SLE) is related to processes like apoptosis and inflammation [28,29]. Expression of MALAT1 in patients with RA is related to disease activity and in SLE nephritis is considered as a potential discriminatory biomarker [30,31]. MALAT1 is thought to take part in the progression of fibrosis in several ways. It can stimulate fibroblast proliferation and promote disbalance in the extracellular matrix by increasing the expression of collagen or inhibiting the production of matrix metalloproteinase enzymes. Additionally, MALAT1 participates in the epithelial-mesenchymal transformation, a major component of the fibrotic process [32]. MALAT1 overexpression is related to different types of lung injury and fibrosis development, by mainly endorsing the production of cytokines with pro-inflammatory and pro-fibrotic effects [32]. However, MALAT1 is shown to regulate differential activation of macrophages and response to lung injury. It promotes the pro-inflammatory M1 phenotype and suppresses the anti-fibrotic M2 phenotype of macrophages [33]. Lung fibrosis, presented by the development of pulmonary arterial hypertension (PAH) is the leading cause of mortality among SSc patients [34], and increased levels of MALAT could be associated with it.

The second lncRNA identified in our study, NEAT1, is known for its involvement in tumorigenesis, mainly acting as a competing endogenous RNA for tumor-suppressor miRNAs, thus, inducing cancer development [35]. It is also proposed that NEAT1 takes part in inflammation as well as in autoimmune response. For example, it activates different inflammasome complexes in LPS stimulated murine macrophages [22]. The lncRNA is found to be increased in serum of patients with multiple sclerosis and is considered to regulate IL-8 expression [36]. In RA patients NEAT1 is upregulated in blood mononuclear cells and serum exosomes, where it is believed to play a role in the pathogenesis of RA [30]. NEAT1 is increased in PBMCs of patients with SLE where it is associated with the regulation of IL-6 and CXCL10. NEAT1 expression is induced by LPS stimulation and regulated through p38. It next controls the production of inflammatory mediators throughout the MAPK pathway [37]. Here we present novel data regarding the expression of NEAT in patients with SSc.

Members of the miR-30 family are described as players in normal cell development and disease [38]. Besides their well-defined role as oncogenic or tumor suppressive molecules, a growing body of evidence shows their involvement in the pathophysiology of autoimmune diseases. The different miR-30 family members are found to be up- or down- regulated in patients with type 1 diabetes mellitus, SLE, RA, myasthenia gravis, multiple sclerosis and SSc [39,40,41,42,43] and their expression is related to disease development or progression. Data from these studies, reveal that members of the miR-30 family have differential expression patterns in various diseases. Participation of miR-30e-5p in inflammatory conditions is under observation. The limited data available show a possible contribution to neuroinflammation in Parkinson’s diseases and innate immune responses during virus infections and SLE [44,45].

Our current results demonstrate the existence of opposite expression patterns between MALAT1/NEAT1 (upregulation) and miR-30e-5p (downregulation) in WBCs of patients with SSc. In addition, we found that plasma levels of miR-30a-5p were also decreased in patients. The two miRNAs share the same seed sequence that might result in their sequestration by lncRNAs. The reverse correlation in the expression profiles of these molecules could be explained by the action of MALAT1 and NEAT1 as a competitive endogenous RNA that functions as a decoy for miR-30-5p. Molecular interactions between MALAT1 and the miR-30 family are based on the presence of a complementary sequence in the lncRNA with the seed region of miRNAs. The functional interplay between MALAT1 and miR-30 family members, as well as between miR-30a-5p and CHI3L1 has been proven in several reports. MALAT1 was shown to sponge miR-30-5p miRNAs in different processes. Upregulation of MALAT1 downregulates miR-30a-5p and endorses Beclin1 dependent autophagy in primary cerebral cortex neuronal cultures obtained from 17-days-old mice and thus, exacerbates neuronal cell death after cerebral ischemic injury. In this model, suppression of MALAT1 results in a decrease of Beclin1 levels and in the improvement of congenital behavior, suggesting that MALAT1 could be a therapeutic target [46]. During hippocampal neurite outgrowth and osteoblast differentiation MALAT1 expression is increased which promotes these two processes [47] The authors showed that miR-30 was a sponge target of MALAT1 and MALAT1/miR-30 altered neurite outgrowth in hippocampal neurons, as revealed by luciferase reporter assay, performed in HT22 and COS1 cells. Inhibitory effects of miR-30 on spastin luciferase activity, mRNA and protein expression were abolished by MALAT1, indicating that spastin was a downstream effector of MALAT1/miR-30 [47]. In addition, the interaction between MALAT1 and miR-30 family members has been shown to promote osteoblast differentiation of adipose-derived mesenchymal stem cells by increasing Runx2 expression [48]. Bifluorescein report experiments performed in 293T cells, confirmed that miR-30 is a potential target of MALAT1 and Runx2 is a potential target of miR-30 [48]. Altogether, the data underlined the diverse and cell-specific effects of the MALAT1/miR-30-5p interaction. According to our knowledge, no data are present about the interaction between NEAT1 and miR-30-5p family members.

**Figure 6:**
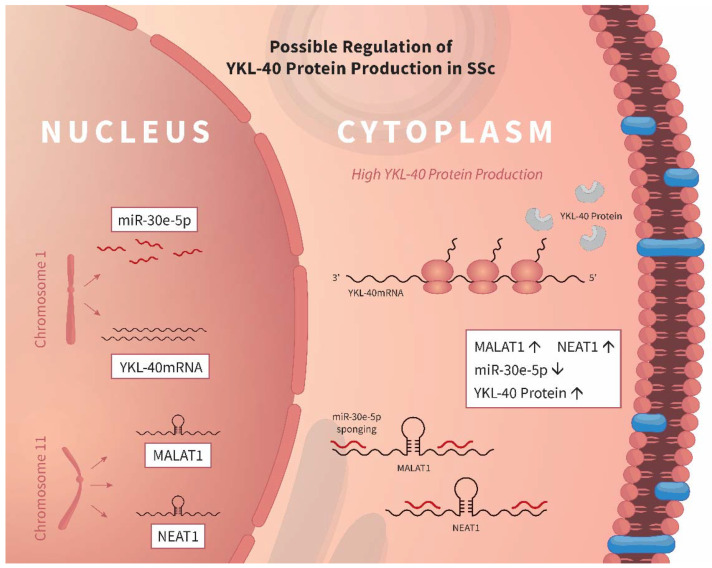
Possible lncRNAs/miR30e/CHI3L1regulatory axis in SSc. In SSc, the expression of MALAT1 and NEAT1 in WBCs is upregulated. MALAT1 and NEAT1 can act as competitive endogenous RNAs for miR-30e. Interaction between MALAT1/NEAT1 and miR-30e-5p would abolish the suppressive effect of miR-30e-5p over CHI3L1production, leading to increased serum levels of the glycoprotein in patients with SSc. ↑ indicates induction, whereas ↓ shows downregulation.

Our results indicate a possible regulatory axis of CHI3L1 synthesis–lncRNAs/miR30e/CHI3L1. We suggest that MALAT1/NEAT1 sponges miR-30e-5p in the WBCs of SSc patients which in turn promotes increased production of the CHI3L1protein. The proposed mechanism for CHI3L1 control in SSc is presented in Figure 6. The potential action of MALAT1 over miR-30e-5, but not on other miR-30 family members, despite the similarity in the seed region of miRNAs, could be due to the presence of different compensatory sequences located near the 3′end, which endow them with distinct biological behaviors and ability to interact with other RNA molecules [38].

MiR-30a-5p downregulation in plasma could be caused by a similar pattern, as MALAT1 is proven to exert its function as a miRNA sponge in a medium, without affecting cell expression levels of miRNAs [49]. Decreased expression of miR-30a-5p is detected in the dermal fibroblasts in patients with SSc. Its suppression is related to increased levels of B-cell activating factor (BAFF), which is associated with an increased risk of the development of an autoimmune response [50]. Direct interaction between miR-30a-5p and CHI3L1 mRNA is observed in human pulmonary artery endothelial cells (HPAEC) under hypoxic conditions in vitro. Expression of miR-30a-5p does not affect CHI3L1 mRNA levels, but a decrease in protein synthesis is detected [51]. This in vitro model represents ischemic injury in PAH which is the most serious complication in SSc [34]. Another miR-30 family member–miR-30b is also found to be downregulated in sera from SSc patients, as well as in the skin of bleomycin-induced mouse models of SSc. Levels of miR-30b were inversely correlated with modified Rodnan skin scores of patients, reflecting disease severity [52]. The decline in circulating miR-30a-5p levels could also be used as a discriminative marker in SSc diagnosis. miRNAs are secreted into the bloodstream as miRNA-protein complexes, or in exosomes which contribute to the high level of stability under a wide range of conditions [53]. Therefore, these molecules seem to be suitable for application as biomarkers.

However, there are some limitations of the present study. The relatively small number of patients enrolled makes it not representative enough. Additionally, all patients and controls are females as the disease is predominantly associated with the female gender. Moreover, SSc patients received different medications which may cause bias in the evaluation of the results. Our suggestion for the interplay between MALAT1, miR-30 family members and CHI3L1 is based on the results from the prediction analysis and the observed changes in their expression profile and from CHI3L1protein levels. Future in vitro experiments for the direct interaction between these molecules are needed to verify further our hypothesis.

## 5. Conclusions

Increased levels of MALAT1 and NEAT1 could be associated with the downregulation of miR-30e-5p and miR-30a-5p expression in WBCs and plasma in SSc. We hypothesize that a regulatory axis exists in WBCs of SSc patients in which there is an interplay between lncRNAs/miR30e/CHI3L1. MALAT1 and NEAT1 could possibly act as miR-30e-5p and miR-30a-5p decoys. As a result, high serum protein levels of CHI3L1 in SSc patients are detected. In conclusion, novel data for a regulatory axis based on interactions of lncRNA, miRNA and the pro-inflammatory glycoprotein in SSc are presented.

## Data Availability

The data presented in this study are available on request from the corresponding author. The data are not publicly available due to privacy restrictions.

## Supplementary Materials

The following supporting information can be downloaded at: www.mdpi.com/article/10.3390/biomedicines10020496/s1. Table S1: Demographic and clinical characteristics of patients and controls.

## References

[1] Asano Y. (2020). The Pathogenesis of Systemic Sclerosis: An Understanding Based on a Common Pathologic Cascade across Multiple Organs and Additional Organ-Specific Pathologies. J. Clin. Med..

[2] Orlandi M., Barsotti S., Lepri G., Codullo V., Di Battista M., Guiducci S., Della Rossa A. (2018). One year in review 2018: Systemic sclerosis. Clin. Exp. Rheumatol..

[3] Dolcino M., Tinazzi E., Puccetti A., Lunardi C. (2019). In Systemic Sclerosis, a Unique Long Non Coding RNA Regulates Genes and Pathways Involved in the Three Main Features of the Disease (Vasculopathy, Fibrosis and Autoimmunity) and in Carcinogenesis. J. Clin. Med..

[4] Mazzone R., Zwergel C., Artico M., Taurone S., Ralli M., Greco A., Mai A. (2019). The emerging role of epigenetics in human autoimmune disorders. Clin. Epigenetics.

[5] Surace A.E.A., Hedrich C.M. (2019). The Role of Epigenetics in Autoimmune/Inflammatory Disease. Front. Immunol..

[6] Statello L., Guo C.J., Chen L.L., Huarte M. (2021). Gene regulation by long non-coding RNAs and its biological functions. Nat. Rev. Mol. Cell Biol..

[7] Eulalio A., Huntzinger E., Izaurralde E. (2008). Getting to the root of miRNA-mediated gene silencing. Cell.

[8] Sohel M.H. (2016). Extracellular/Circulating MicroRNAs: Release Mechanisms, Functions and Challenges. Achiev. Life Sci..

[9] Zhao T., Su Z., Li Y., Zhang X., You Q. (2020). Chitinase-3 like-protein-1 function and its role in diseases. Signal. Transduct. Target Ther..

[10] Yeo I.J., Lee C.K., Han S.B., Yun J., Hong J.T. (2019). Roles of chitinase 3-like 1 in the development of cancer, neurodegenerative diseases, and inflammatory diseases. Pharmacol. Ther..

[11] Kazakova M., Sarafian V. (2013). YKL-40 in health and disease: A challenge for joint inflammation. Biomed. Rev..

[12] Dichev V., Mehterov N.H., Kazakova M.H., Karalilova R.V., Batalov A.Z., Sarafian V.S. (2021). Serum protein levels of YKL-40 and plasma miR-214 expression in patients with systemic sclerosis. Mod. Rheumatol..

[13] van den Hoogen F., Khanna D., Fransen J., Johnson S.R., Baron M., Tyndall A., Matucci-Cerinic M., Naden R.P., Medsger T.A., Carreira P.E. (2013). 2013 classification criteria for systemic sclerosis: An American college of rheumatology/European league against rheumatism collaborative initiative. Ann. Rheum. Dis..

[14] LeRoy E.C., Medsger T.A. (2001). Criteria for the classification of early systemic sclerosis. J. Rheumatol..

[15] Betel D., Wilson M., Gabow A., Marks D.S., Sander C. (2008). The microRNA.org resource: Targets and expression. Nucleic Acids Res..

[16] McGeary S.E., Lin K.S., Shi C.Y., Pham T.M., Bisaria N., Kelley G.M., Bartel D.P. (2019). The biochemical basis of microRNA targeting efficacy. Science.

[17] Vejnar C.E., Zdobnov E.M. (2012). MiRmap: Comprehensive prediction of microRNA target repression strength. Nucleic Acids Res..

[18] Karagkouni D., Paraskevopoulou M.D., Tastsoglou S., Skoufos G., Karavangeli A., Pierros V., Zacharopoulou E., Hatzigeorgiou A.G. (2020). DIANA-LncBase v3: Indexing experimentally supported miRNA targets on non-coding transcripts. Nucleic Acids Res..

[19] Paraskevopoulou M.D., Karagkouni D., Vlachos I.S., Tastsoglou S., Hatzigeorgiou A.G. (2018). microCLIP super learning framework uncovers functional transcriptome-wide miRNA interactions. Nat. Commun..

[20] Li J.H., Liu S., Zhou H., Qu L.H., Yang J.H. (2014). starBase v2.0: Decoding miRNA-ceRNA, miRNA-ncRNA and protein-RNA interaction networks from large-scale CLIP-Seq data. Nucleic Acids Res..

[21] Biswas S., Thomas A.A., Chen S., Aref-Eshghi E., Feng B., Gonder J., Sadikovic B., Chakrabarti S. (2018). MALAT1: An Epigenetic Regulator of Inflammation in Diabetic Retinopathy. Sci. Rep..

[22] Zhang P., Cao L., Zhou R., Yang X., Wu M. (2019). The lncRNA Neat1 promotes activation of inflammasomes in macrophages. Nat. Commun..

[23] Chen Y., Fu Y., Song Y.F., Li N. (2019). Increased Expression of lncRNA UCA1 and HULC Is Required for Pro-inflammatory Response During LPS Induced Sepsis in Endothelial Cells. Front. Physiol..

[24] Luo Y., Wang Y., Shu Y., Lu Q., Xiao R. (2015). Epigenetic mechanisms: An emerging role in pathogenesis and its therapeutic potential in systemic sclerosis. Int. J. Biochem. Cell Biol..

[25] Di Marco M., Ramassone A., Pagotto S., Anastasiadou E., Veronese A., Visone R. (2018). MicroRNAs in Autoimmunity and Hematological Malignancies. Int. J. Mol. Sci..

[26] Zou Y., Xu H. (2020). Involvement of long noncoding RNAs in the pathogenesis of autoimmune diseases. J. Transl. Autoimmun..

[27] Karalilova R., Kazakova M., Sapundzhieva T., Dichev V., Batalov Z., Sarafian V., Batalov A. (2019). Serum YKL-40 and IL-6 levels correlate with ultrasound findings of articular and periarticular involvement in patients with systemic sclerosis. Rheumatol. Int..

[28] Pan F., Zhu L., Lv H., Pei C. (2016). Quercetin promotes the apoptosis of fibroblast-like synoviocytes in rheumatoid arthritis by upregulating lncRNA MALAT1. Int. J. Mol. Med..

[29] Yang H., Liang N., Wang M., Fei Y., Sun J., Li Z., Xu Y., Guo C., Cao Z., Li S. (2017). Long noncoding RNA MALAT-1 is a novel inflammatory regulator in human systemic lupus erythematosus. Oncotarget.

[30] Song J., Kim D., Han J., Kim Y., Lee M., Jin E.J. (2015). PBMC and exosome-derived Hotair is a critical regulator and potent marker for rheumatoid arthritis. Clin. Exp. Med..

[31] Mihaylova G., Vasilev V., Kosturkova M.B., Stoyanov G.S., Radanova M. (2020). Long Non-Coding RNAs as New Biomarkers in Lupus Nephritis: A Connection Between Present and Future. Cureus.

[32] Li Y., Liu F., Cai Y., Yang Y., Wang Y. (2021). LncRNA MALAT1: A Potential Fibrosis Biomarker and Therapeutic Target. Crystals.

[33] Cui H., Banerjee S., Guo S., Xie N., Ge J., Jiang D., Zornig M., Thannickal V.J., Liu G. (2019). Long noncoding RNA Malat1 regulates differential activation of macrophages and response to lung injury. JCI Insight..

[34] Bruni C.G., Guignabert C., Manetti M., Cerinic M., Humbert H. (2021). The multifaceted problem of pulmonary arterial hypertension in systemic sclerosis. Lancet Rheumatol..

[35] Klec C., Prinz F., Pichler M. (2019). Involvement of the long noncoding RNA NEAT1 in carcinogenesis. Mol. Oncol..

[36] Santoro M., Nociti V., Lucchini M., De Fino C., Losavio F.A., Mirabella M. (2016). Expression Profile of Long Non-Coding RNAs in Serum of Patients with Multiple Sclerosis. J. Mol. Neurosci..

[37] Zhang F., Wu L., Qian J., Qu B., Xia S., La T., Wu Y., Ma J., Zeng J., Guo Q. (2016). Identification of the long noncoding RNA NEAT1 as a novel inflammatory regulator acting through MAPK pathway in human lupus. J. Autoimmun..

[38] Mao L., Liu S., Hu L., Jia L., Wang H., Guo M., Chen C., Liu Y., Xu L. (2018). miR-30 Family: A Promising Regulator in Development and Disease. Biomed. Res. Int..

[39] Dieter C., Assmann T.S., Costa A.R., Canani L.H., de Souza B.M., Bauer A.C., Crispim D. (2019). MiR-30e-5p and MiR-15a-5p Expressions in Plasma and Urine of Type 1 Diabetic Patients With Diabetic Kidney Disease. Front. Genet..

[40] Fang X., Sun D., Wang Z., Yu Z., Liu W., Pu Y., Wang D., Huang A., Liu M., Xiang Z. (2017). MiR-30a Positively Regulates the Inflammatory Response of Microglia in Experimental Autoimmune Encephalomyelitis. Neurosci. Bull..

[41] Kim B.S., Jung J.Y., Jeon J.Y., Kim H.A., Suh C.H. (2016). Circulating hsa-miR-30e-5p, hsa-miR-92a-3p, and hsa-miR-223-3p may be novel biomarkers in systemic lupus erythematosus. HLA.

[42] Liu Y., Dong J., Mu R., Gao Y., Tan X., Li Y., Li Z., Yang G. (2013). MicroRNA-30a promotes B cell hyperactivity in patients with systemic lupus erythematosus by direct interaction with Lyn. Arthritis Rheum..

[43] Sabre L., Maddison P., Sadalage G., Ambrose P.A., Punga A.R. (2018). Circulating microRNA miR-21-5p, miR-150-5p and miR-30e-5p correlate with clinical status in late onset myasthenia gravis. J. Neuroimmunol..

[44] Li D., Yang H., Ma J., Luo S., Chen S., Gu Q. (2018). MicroRNA-30e regulates neuroinflammation in MPTP model of Parkinson's disease by targeting Nlrp3. Hum. Cell.

[45] Mishra R., Bhattacharya S., Rawat B.S., Kumar A., Kumar A., Niraj K., Chande A., Gandhi P., Khetan D., Aggarwal A. (2020). MicroRNA-30e-5p has an Integrated Role in the Regulation of the Innate Immune Response during Virus Infection and Systemic Lupus Erythematosus. iScience.

[46] Guo D., Ma J., Yan L., Li T., Li Z., Han X., Shui S. (2017). Down-Regulation of Lncrna MALAT1 Attenuates Neuronal Cell Death Through Suppressing Beclin1-Dependent Autophagy by Regulating Mir-30a in Cerebral Ischemic Stroke. Cell Physiol. Biochem..

[47] Jiang T., Cai Z., Ji Z., Zou J., Liang Z., Zhang G., Liang Y., Lin H., Tan M. (2020). The lncRNA MALAT1/miR-30/Spastin Axis Regulates Hippocampal Neurite Outgrowth. Front. Cell Neurosci..

[48] Yi J., Liu D., Xiao J. (2019). LncRNA MALAT1 sponges miR-30 to promote osteoblast differentiation of adipose-derived mesenchymal stem cells by promotion of Runx2 expression. Cell Tissue Res..

[49] Wang L., Liu J., Xie W., Li G., Yao L., Zhang R., Xu B. (2019). Overexpression of MALAT1 Relates to Lung Injury through Sponging miR-425 and Promoting Cell Apoptosis during ARDS. Can. Respir. J..

[50] Alsaleh G., Francois A., Philippe L., Gong Y.Z., Bahram S., Cetin S., Pfeffer S., Gottenberg J.E., Wachsmann D., Georgel P. (2014). MiR-30a-3p negatively regulates BAFF synthesis in systemic sclerosis and rheumatoid arthritis fibroblasts. PLoS ONE.

[51] Tan H., Yao H., Lie Z., Chen G., Lin S., Zhang Y. (2019). MicroRNA30a5p promotes proliferation and inhibits apoptosis of human pulmonary artery endothelial cells under hypoxia by targeting YKL40. Mol. Med. Rep..

[52] Tanaka S., Suto A., Ikeda K., Sanayama Y., Nakagomi D., Iwamoto T., Suzuki K., Kambe N., Matsue H., Matsumura R. (2013). Alteration of circulating miRNAs in SSc: miR-30b regulates the expression of PDGF receptor beta. Rheumatology.

[53] Koberle V., Pleli T., Schmithals C., Augusto Alonso E., Haupenthal J., Bonig H., Peveling-Oberhag J., Biondi R.M., Zeuzem S., Kronenberger B. (2013). Differential stability of cell-free circulating microRNAs: Implications for their utilization as biomarkers. PLoS ONE.

