# Supplementary material for: The lncRNAs/miR-30e/CHI3L1 Axis Is Dysregulated in Systemic Sclerosis
Source: Biomedicines. 2022 Feb 19;10(2):496. doi: 10.3390/biomedicines10020496 (PMC8962397; doi:10.3390/biomedicines10020496)
Supplement: Supplementary file 1 [file biomedicines-10-00496-s001.zip › biomedicines-1604356-supplementary.pdf]

Table S1. Demographic and clinical characteristics of patients and controls.

Variables	Group			Patients group		
	Healthy subjects	SSc patients	*p*	dcSSc patients	lcSSc patients	*p*
N	14	40		23	17	
Age (Mean±SD)	48.3±7.8	48.9±14.5	0.769	52.1±12.0	46±16.1	0.099
ESR mm/h<br>Median (min-max)	<12.0	16.5<br>(4-36)	NA	20.5<br>(7-36)	14<br>(4-21)	0.002**
CRP, mg/L<br>Median (min-max)	<6.0	4.55<br>(1.1-14.8)	NA	8.2<br>(1.1-14.8)	4.1<br>(2.1-13.2)	0.245
mRSS median (min-max)	NA	16.9<br>(8-37)	NA	19.4<br>(8-37)	12.0<br>(9-24)	0.008*
Disease duration - >3 years from first non-RP **n (%)**	NA	32 (80)	NA	17 (74)	0	NA
Capillaroscopy<br>Active **n (%)**<br>Early **n (%)**<br>Late **n (%)**<br>Early/Active **n (%)**	NA<br>NA	24 (60)<br>5 (12.5)<br>8 (20)<br>3 (7.5)		10 (43.6)<br>5 (21.7)<br>5 (21.7)<br>3 (13)	14 (82)<br>0<br>3 (18)<br>0	NA<br>NA<br>NA<br>NA
ANA positivity<br>**n (%)**	NA	34 (85)	NA	23 (100)	11 (64)	NA
ATA **n (%)**<br>ATA/ARA **n (%)**<br>ACA **n (%)**<br>ATA/ACA **n (%)**	NA<br>NA<br>NA<br>NA	7 (20.6)<br>3 (8.8)<br>4 (11.8)<br>3 (8.8)	NA<br>NA<br>NA<br>NA	7 (30.4)<br>0<br>1 (4.3)<br>3 (13)	0<br>3 (17)<br>4 (23)<br>0	NA<br>NA<br>NA<br>NA
Organ involvement<br>**n (%)**	NA	20 (50)	NA	14 (60.9)	6 (35.4)	NA
PAH **n (%)**<br>IHD **n (%)**<br>ILD **n (%)**<br>GIT **n (%)**	NA<br>NA<br>NA<br>NA	2 (5)<br>4 (10)<br>3 (7.5)<br>3 (7.5)	NA<br>NA<br>NA<br>NA	0<br>2 (8.7)<br>3 (13)<br>2 (8.7)	2 (11.8)<br>2 (11.8)<br>0<br>1 (5.9)	NA<br>NA<br>NA<br>NA
Multiple systems<br>**n (%)**	NA	8 (20)	NA	7 (30.5)	1 (5.9)	NA
Lung fibrosis **n (%)**	NA	4 (10)	NA	4 (17.4)	0	NA
Treatment<br>CCB **n (%)**<br>ASA **n (%)**<br>MTX **n (%)**<br>CS **n (%)**<br>AZA **n (%)**<br>Combined **n (%)**	NA<br>NA<br>NA<br>NA<br>NA<br>NA	6 (15)<br>3 (7.5)<br>4 (10)<br>3 (7.5)<br>1 (2.5)<br>23 (57.5)	NA<br>NA<br>NA<br>NA<br>NA<br>NA	1 (4.3)<br>1 (4.3)<br>2 (8.7)<br>3 (13)<br>1 (4.3)<br>15 (65.4)	5 (29.4)<br>2 (11.8)<br>2 (11.8)<br>0<br>0<br>8 (47.1)	NA<br>NA<br>NA<br>NA<br>NA<br>NA
Data for experimental variables						
	Healthy subjects	SSc patients	*p*	dcSSc patients	lcSSc patients	*p*
YKL-40 ng/ml (n)<br>Median (min-max)	(14) 44.52<br>(28.15-70.81)	(40) 76.58<br>(21.64-310.5)	0.004**	(23) 87.1<br>(21.64-310.5)	(17) 68.5<br>(24.32-264.7)	0.119
CHI3L1<br>(n) Median FC (min-	(12) 0.924<br>(0.35-2.23)	(31) 1.358<br>(0.34-5.14)	0.345	(19) 1.153<br>(0.34-2.84)	(12) 1.679<br>(0.8-5.14)	0.08

max)						
MALAT1 (n) Median FC (min-mix)	(12) 1.058 (0.12-2.46)	(31) 1.952 (0.04-6.38)	0.049*	(19) 1.907 (0.04-4.39)	(12) 2.956 (0.796.38)	0.08
NEAT1 (n) Median FC (min-max)	(12) 1.072 (0.18-2.81)	(31) 2.328 (0.34-5.82)	0.008**	(19) 2.471 (0.34-4.79)	(12) 1.993 (0.37-5.82)	0.876
miR-30e (n) Mean FC (SD)	(12) 1.058 (0.338)	(31) 0.799 (0.398)	0.03*	(19) 0.829 (0.392)	(12) 0.765 (0.425)	0.613
miR-30a (n) Median FC (min-max)	(9) 0.972 (0.28-2.23)	(30) 0.411 (0.005-2.95)	0.01*	(16) 0.413 (0.005-1.88)	(14) 0.397 (0.06-2.95)	0.135

Systemic sclerosis – SSc; diffuse cutaneous systemic sclerosis – dcSSc; limited cutaneous systemic sclerosis – lcSSc, non-Rayno Phenomen – non-RP, ANA – anti-nuclear antibodies, ATA – anti-Scl-70 antibodies, ARA – anti-RNA polymerase antibodies, ACA – anti-centromere antibodies, RHF - right heart failure, GIT - gastrointestinal tract, IHD - ischemic heart disease, PAH - pulmonary arterial hypertension, ILD - interstitial lung disease, Calcium channel blockers – CCB, Acetyl-salicylic acid – ASA, Methotrexate – MTX, Corticosteroids – CS, Azathioprin – AZA, * statistical significance $p < 0.05$, ** statistical significance $p < 0.01$.
